# Korean Red ginseng enhances ZBP1-mediated cell death to suppress viral protein expression in host defense against Influenza A virus

**DOI:** 10.71150/jm.2409007

**Published:** 2025-01-24

**Authors:** Jueun Oh, Hayeon Kim, Jihye Lee, Suhyun Kim, Seyun Shin, Young-Eui Kim, Sehee Park, SangJoon Lee

**Affiliations:** 1 Department of Biological Science, Ulsan National Institute of Science and Technology (UNIST), Ulsan 44919, Republic of Korea; 2 Division of Acute Viral Disease, Center for Emerging Virus Research, National Institute of Infectious Diseases, Korea National Institute of Health, Cheongju 28159, Republic of Korea; 3 Graduate School of Health Science and Technology, Ulsan National Institute of Science and Technology (UNIST), Ulsan 44919, Republic of Korea

**Keywords:** cell death, host defense, influenza A virus, Red ginseng, ZBP1

## Abstract

Korean Red ginseng has emerged as a potent candidate in the fight against various viral infections, demonstrating significant efficacy both in vitro and in vivo, particularly against influenza A viruses. Despite substantial evidence of its antiviral properties, the detailed molecular mechanisms through which it reduces viral lethality remain insufficiently understood. Our investigations have highlighted the superior effectiveness of Korean Red ginseng against influenza viruses, outperforming its effects on numerous other viral strains. We aim to uncover the specific mechanisms by which Korean Red ginseng exerts its antiviral effects, focusing on influenza A viruses. Our prior studies have identified the role of Z-DNA-binding protein 1 (ZBP1), a signaling complex involved in inducing programmed cell death in response to influenza virus infection. Given the critical role of ZBP1 as a sensor for viral nucleic acid, we hypothesize that Korean Red ginseng may modulate the ZBP1-derived cell death pathway. This interaction is anticipated to enhance cell death while concurrently suppressing viral protein expression, offering novel insights into the antiviral mechanism of Korean Red ginseng against influenza A viruses.

## Introduction

Korean Red ginseng is a thermally processed form of ginseng produced by repeatedly steaming and naturally drying fresh ginseng (Yun, 2001). This method induces alterations in chemical constituents, such as ginsenosides Rg2, Rg3, Rh1, and Rh2, resulting in Korean Red ginseng exhibiting enhanced pharmacological efficacy and stability relative to fresh ginseng (Kim et al., 2000). Numerous pharmacological benefits of ginseng and its components have been documented, including, anti-cancer (Qian et al., 2014; Sun et al., 2011; Yue et al., 2006; Yun, 2003), anti-allergic (Jung et al., 2013; Kim & Yang, 2011; Park et al., 2004; Sumiyoshi et al., 2010), anti-inflammatory (Ahn et al., 2006; Lee & Cho, 2011; Park et al., 2004, 2009; Zhao et al., 2024), anti-fatigue (Bao et al., 2016; Choi et al., 2011; Tan et al., 2013; Wang et al., 2010), immunomodulatory effects (Kenarova et al., 1990; Kim et al., 1990; Nakaya et al., 2004; Sung et al., 2005) and tumor cell growth inhibition (Ota et al., 1987, 1996; Tode et al., 1993), and cell death (Li et al., 2011; Park et al., 2012; Popovich & Kitts, 2002; Wakabayashi et al., 1998). Furthermore, the antiviral properties of ginseng have been substantiated, with Korean Red ginseng extracts and ginsenosides demonstrating antiviral activities against influenza virus (Lee et al., 2014a; Yoo et al., 2012a, 2012b), rhinovirus (Kim et al., 2024; Song et al., 2014), human immunodeficiency virus (Cho & Kim, 2017; Sung et al., 2005, 2009).

In particular, the antiviral properties of Korean Red ginseng play a crucial role in supporting the innate immune system. Innate immunity is the first line of defense activated when pathogens, such as viruses, invade the host. The host continuously maintains defensive and offensive mechanisms against pathogens of varying sizes and lethality. The innate immune process can be broadly divided into three stages with pathogen recognition, activation of signaling pathways, and establishment of an antiviral state. Upon pathogen recognition, signaling pathways are activated, leading to the induction of an antiviral state. Pathogen recognition is mediated by pattern recognition receptors (PRRs), which detect pathogen-associated molecular patterns (PAMPs) and damage-associated molecular patterns (DAMPs). Prominent PRRs include Toll-like receptors (TLRs), retinoic acid-inducible gene I (RIG-I)-like receptors (RLRs), nucleotide-binding oligomerization domain (NOD)-like receptor family proteins (NLRs), absent in melanoma 2 (AIM2), and Z-DNA binding protein 1 (ZBP1). This process involves the recruitment of immune cells such as macrophages and neutrophils, as well as the activation of programmed cell death (PCD) pathways to eliminate infected cells and inhibit viral spread (Ashida et al., 2011; Fink & Cookson, 2005; Lamkanfi & Dixit, 2010; Lee et al., 2018, 2019).

ZBP1, also referred to as DAI or DLM-1, is a nucleic acid sensor characterized by the presence of two Zα domains that bind to Z-DNA and Z-RNA (Ha et al., 2008; Maelfait et al., 2017; Placido et al., 2007; Schwartz et al., 2001; Thapa et al., 2016). ZBP1 plays a crucial role in mediating host defense against a variety of viruses through the recognition of viral nucleic acids. Recent research has identified ZBP1 as a central regulator of PANoptosis (Banoth et al., 2020; Karki et al., 2021b; Lee et al., 2021c; Oh & Lee, 2023), a unique form of programmed cell death essential for the defense against viral infections, including SARS-CoV-2 (Karki et al., 2021a, 2022; Zheng et al., 2020) and Influenza A virus (IAV) (Kesavardhana et al., 2020; Kuriakose et al., 2016; Thapa et al., 2016).

Korean Red ginseng has demonstrated significant efficacy against various viral infections (Lee et al., 2013, 2014b; Song et al., 2014), particularly influenza viruses, in both in vitro and in vivo studies. Despite the substantial evidence supporting its antiviral properties, the detailed molecular mechanisms underlying its reduction of viral lethality remain insufficiently elucidated. Here, we discovered that Korean Red ginseng modulates the ZBP1-derived cell death pathway, a mechanism hypothesized to enhance programmed cell death while simultaneously suppressing viral protein expression.

In our study, wild-type (WT) bone marrow-derived macrophages (BMDMs) infected with influenza A virus and treated with Korean Red ginseng exhibited increased cell death and reduced viral protein expression compared to untreated controls. This suggests that Korean Red ginseng enhances the ZBP1-derived cell death pathway. In contrast, *Zbp1*^–/–^ BMDMs infected with the virus showed significantly reduced cell death and increased viral protein expression, indicating the critical role of ZBP1 in mediating this response.

To further investigate these findings, WT and *Zbp1*^–/–^ mice were administered Korean Red ginseng for 7 days prior to being challenged with influenza A virus for 14 days. WT mice treated with Korean Red ginseng demonstrated improved survival rates compared to those infected with the virus alone. However, *Zbp1*^–/–^ mice showed no significant differences in survival across all groups, reinforcing the role of ZBP1 in the antiviral effects of Korean Red ginseng. These results confirm that Korean Red ginseng enhances the ZBP1-derived cell death pathway, providing novel insights into its antiviral mechanism against influenza viruses.

## Materials and Methods

### Mice

C57BL/6J (wild type [WT]) (Hyochang Science) and *Zbp1*^–/–^ (Cyagen) mice were purchased from the indicated sources. The mice were group-housed (up to five mice per cage), bred under standard pathogen-free housing conditions in the animal facility of Ulsan National Institute of Science and Technology (UNIST) under a 12 h light/dark cycle (lights on from 7 A.M. to 7 P.M.), and fed standard chow. Both male and female mice were used in this study. Age- and sex-matched 6- to 8-week-old mice were used for the in vivo studies, and 6- to 12-week-old mice were used for the in vitro studies. Cohoused animals were used for in vivo analyses. All the experimental procedures were conducted in accordance with protocols that were approved by the Institutional Animal Care and Utilization Committee of UNIST.

### Cell culture

Primary bone marrow-derived macrophages (BMDMs) were cultivated for six days in IMDM (Thermo Fisher Scientific, 12440061) supplemented with 5% FBS (Thermo Fisher Scientific, 16000044), 30% L929-conditioned medium, 1% nonessential amino acids (Thermo Fisher Scientific, 11140-050), and 1% penicillin and streptomycin (Thermo Fisher Scientific, 15070-063). BMDMs were seeded in 12-well plates at a density of 1 million cells/well and incubated overnight before use. L929 (ATCC, CCL-1) were purchased and grown in IMDM supplemented with 10% FBS (Thermo Fisher Scientific, 16000044), 1% nonessential amino acids, and 1% penicillin and streptomycin. A549 (gift from Dr. Atsushi Kawaguchi [University of Tsukuba]) were grown in DMEM (Thermo Fisher Scientific, 11995081) supplemented with 5% FBS and 1% penicillin and streptomycin.

### Virus culture

Influenza A virus A/Puerto Rico/8/34 (H1N1, PR8), A/Switzerland/9715293/2013 (H3N2, Switzerland/2013), and A/WSN/1933 (H1N1, WSN) was kindly gifted by Dr. Man-Seong Park (Korea University) and propagated in 11-day-old embryonated chicken eggs by allantoic inoculation. Human herpes simplex virus 1 (HF strain) (ATCC; VR-260) was purchased and propagated in Vero cells (Korean Cell Line Bank; 10081); the virus titer was measured using the plaque assay in Vero cells. Vaccinia virus (Western Reserve, WR) (ATCC; VR-1354) was purchased and propagated in Vero cells (Korean Cell Line Bank; 10081); the virus titer was measured using the plaque assay in Vero cells. Mumps rubulavirus (MuV/Iowa.US/2006) (ATCC; VR-1899) was purchased and propagated in Vero cells; the virus titer was measured using the plaque assay in Vero cells. HCoV-NL63 was purchased in KBPV (KBPV-VR-88) and propagated in Vero cells (Korean Cell Line Bank; 10081); the virus titer was measured using the plaque assay in Vero cells.

### Cell infection and stimulation

For HSV-1 (30 h; MOI 10), VACV (30 h; MOI 10), HCoV-NL63 (30 h; MOI 0.3), and MuV (30 h; MOI 10) infection, cells were infected in DMEM high glucose (Gibco, 11995-065). For IAV (15 h or 30 h; MOI 10) infection, cells were infected in high-glucose DMEM (WELGENE, LM001-03). Korean Red ginseng extracts are provided by Korea Ginseng Corporation (KGC) are added to cells after 1 h of infection.

### Real-time cell death analysis

An IncuCyte S3 imaging system (Sartorius) was purchased, and real-time cell death analysis was performed as previously using IncuCyte S3 imaging system (Essen Biosciences). BMDMs were seeded in 12-well plates (10^6^ cells/well) and stimulated. After incubation, SYTOX Green (Thermo Fisher Scientific, S7020) was added according to the manufacturer’s protocol. Images were acquired every 1 h at 37°C in 5% CO_2_. The resulting images were analyzed using the software supplied with the IncuCyte imager, the number of SYTOX Green-positive BMDM nuclei (Sytox^+^ BMDM nuclei) and SYTOX Green-positive A549 nuclei (Sytox^+^ A549 nuclei) present in each image were counted.

### Immunoblot analysis and quantification

Immunoblotting was performed as previously described (Karki et al., 2022; Lee et al., 2021b; Oh et al., 2023; Wang et al., 2021a, 2022a). Briefly, for caspase analysis, BMDMs were lysed along with their supernatant using 50 μl caspase lysis buffer (1×protease inhibitors, 1× phosphatase inhibitors, 10% NP-40, and 25 mM DTT), followed by the addition of 100 μl 4 × SDS loading buffer. For signalling protein analysis, BMDM supernatants were removed at the indicated time points, the cells were washed once with PBS, and the cells were then lysed with RIPA buffer. Proteins were separated by electrophoresis in 8–15% polyacrylamide gels. After the electrophoretic transfer of the proteins to PVDF membranes (Millipore, IPVH00010), nonspecific binding was blocked by incubation with 5% skim milk. The membranes were then incubated with the following primary antibodies: Influenza A NP Polyclonal Antibody (invitrogen, #PA5-32242, 1:2000), Anti-SARS-CoV-1/2 NP Antibody (Sigma-Aldrich, ZMS1075, 1:2000), Anti-Mumps NP antibody (Abcam, ab9880, 1:2000), Anti-Vaccinia virus antibody (Abcam, ab35219, 1:2000), Anti-HSV-1 ICP8 Major DNA binding protein antibody (Abcam, ab20194, 1:2000), anti-ZBP1 (AdipoGen, AG-20B-0010, 1:2000), and anti-β-actin (Proteintech, 66009-1- IG, 1:5000) antibodies. The membranes were then washed and incubated with the appropriate horseradish peroxidase (HRP)-conjugated secondary antibodies (1:5000 dilution; Jackson Immuno Research Laboratories, anti-rabbit [111-035-047], anti-mouse [315-035-047] antibodies) for 1 h. The proteins were visualized using Luminata Forte Western HRP Substrate (Millipore, WBLUF0500), and the membranes were analyzed using Amersham ImageQuant 800 UV. Images were analyzed by ImageJ. The relative intensity of protein band is quantified by Cytiva ImageQuant™ TL.

### In vivo study of ginseng extracts reduce survival in IAV infection

Age- and sex-matched, 6- to 8-week-old wild-type and *Zbp1*^–/–^ mice were used for infections. For IAV PR8 infection, mice were anaesthetized with 250 mg/kg avertin and then infected intranasally with IAV PR8 in 50 μl PBS containing around 3.04 × 10^3^ PFU. Infected mice were monitored over a period of 14 days for survival. Korean Red ginseng extracts are treated orally 30 mg/kg/day for 7 days before virus infection and after 14 days of virus infection. Lung samples collected at 5 days after infection are fixed at NBF and then H&E staining were conducted through LabCore Korea. The specimens were examined utilizing a DMi8 Inverted microscope (Leica).

## Results

### Korean Red ginseng lowers viral protein expression in BMDMs infected with Influenza A virus

To evaluate the efficacy of Korean Red ginseng in viral clearance against a spectrum of DNA and RNA viruses. Prior to assessing its antiviral properties, the toxicity of Korean Red ginseng on host cells was examined to ensure safe experimental conditions. Wild-type (WT) bone marrow-derived macrophages (BMDMs) (Fig. S1A-B) and A549 (Fig. S1C-D) were treated with varying concentrations of Korean Red ginseng. The concentrations ranged from 25 μg/ml to 2.5 mg/ml, with a tenfold increase between each concentration. Cell viability was assessed after 24 h of treatment. The results revealed no significant difference in cell death between concentrations of 25 μg/ml and 250 μg/ml in both WT BMDMs and A549. However, a notable increase in cell death was observed at concentrations of 25 μg/ml and 2.5 mg/ml in WT BMDMs. Based on these findings, 250 μg/ml was selected as the experimental condition for further investigations (Fig. S1A-D).

**Fig. 1.**
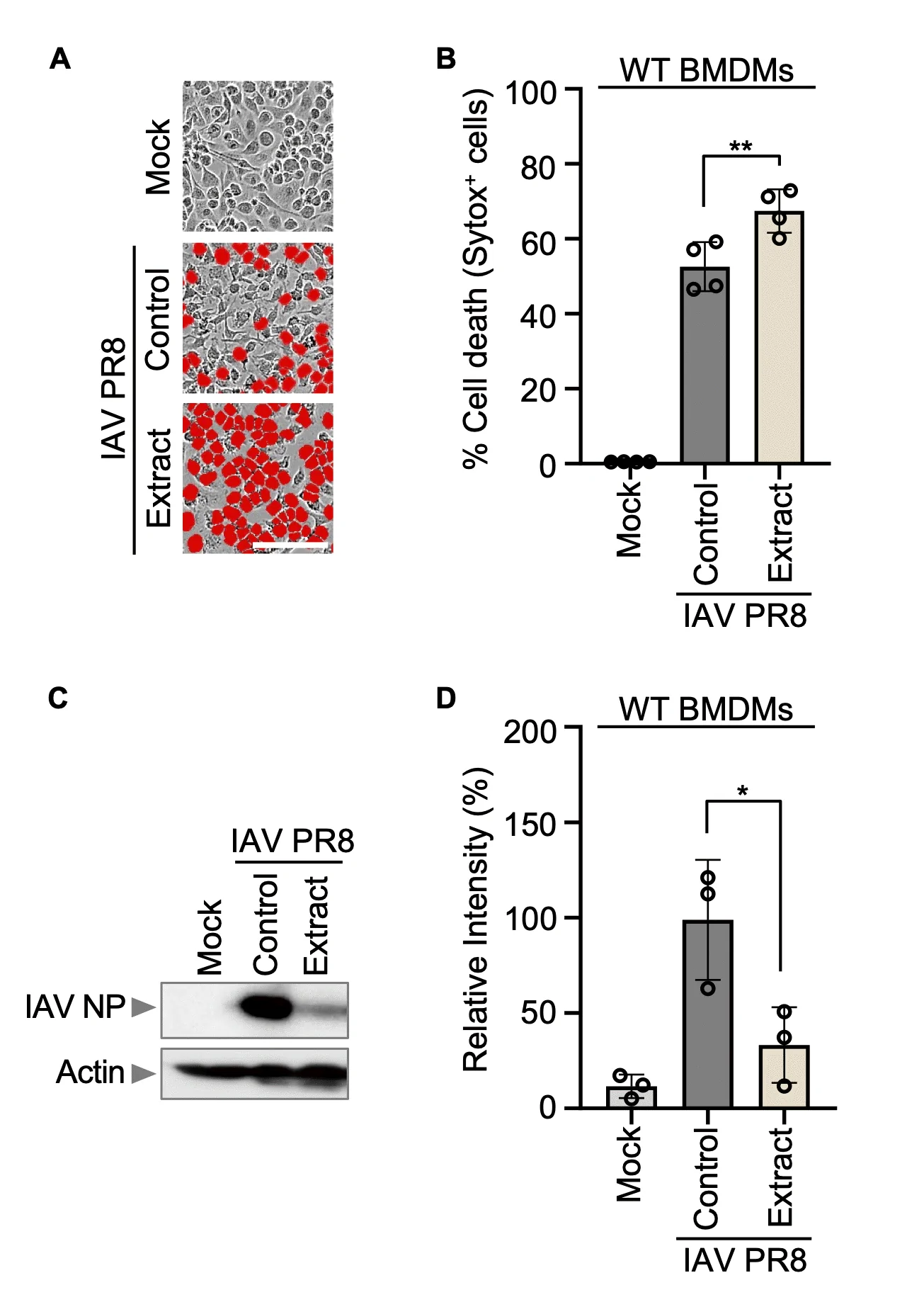
Korean Red ginseng extracts reduce the expression of viral protein NP with inducing cell death in influenza A virus infection. (A) Cell death in wild-type (WT) bone marrow-derived macrophages (BMDMs) after IAV PR8 infection and Korean Red ginseng extracts treatment for 30 h. Red indicates dead cells. Scale bar, 100 μm. Images are representative of at least three independent experiments. (B) Quantification of the cell death in panel A. Data are mean±s.e.m. ^****^P<0.0001 (one-way ANOVA with Dunnett’s multiple comparisons test; n=4 from four biologically independent samples). (C) Immunoblot analysis of IAV NP expression in BMDMs after IAV PR8 infection and Korean Red ginseng extracts treatment. Data are representative of at least three independent experiments. (D) Quantification of relative intensity in panel C. The value of intensity is expression of IAV NP relative to actin in panel C. Data are mean±s.e.m. ^*^P<0.05 (one-way ANOVA with Dunnett’s multiple comparisons test; n=3 from three biologically independent samples).

Among various viruses studied, Korean Red ginseng demonstrated significant efficacy against the influenza virus, particularly in terms of viral clearance (Lee et al., 2014a; Yoo et al., 2012a, 2012b). Initially, WT BMDMs were exposed to the Influenza A virus A/Puerto Rico/8/34 (PR8) at an MOI of 10. Following this, fetal bovine serum (FBS) was introduced one hour later, and subsequently, Korean Red ginseng extracts were administered at a concentration of 250 μg/ml. The incubation period lasted for 30 h. Observations indicated a higher rate of BMDMs cell death in the group treated with Korean Red ginseng compared to control group that was infected with the virus alone (Fig. 1A-B). However, intriguingly, despite the increased cell death, the expression level of viral protein in the Korean Red ginseng-treated group was notably lower compared to the control group infected solely with the virus (Fig. 1C-D). This suggests that while Korean Red ginseng induced cell death in virus infected cells, it simultaneously lowered the expression level of viral protein, ultimately contributing to its clearance.

### Korean Red ginseng specifically shows the efficacy of viral protein expression against influenza viruses

To investigate the efficacy against RNA viruses, human coronavirus (HCoV-NL63; strain: NL63) and mumps virus (MuV; strain: MuV/Iowa.US/2006), were introduced to A549 at an MOI of 10. Following this, FBS was added one hour later to inactivate virus, with Korean Red ginseng treatment commencing another hour after that. Korean Red ginseng extracts was administered at a concentration of 250 μg/ml, and the incubation period lasted for 30 h. For evaluating the results, both host cell death and viral protein expression levels were compared between the Korean Red ginseng-treated groups and the control groups. However, no significant difference was observed between them (Fig. S2A-F). As a result, it was concluded that Korean Red ginseng did not exhibit efficacy of viral protein expression against these RNA viruses.

To investigate the potential virus clearance efficacy of Korean Red ginseng against DNA viruses, an experiment was conducted under conditions identical to the previous RNA virus experiments. Initially, WT BMDMs and *Zbp1*^–/–^ BMDMs were infected with herpes simplex virus 1 (HSV-1; strain: HF) and vaccinia virus (VACV; strain: Western Reserve) at an MOI of 10. Subsequently, FBS was added one hour later, followed by the administration of 250 μg/ml of Korean Red ginseng extracts. The cells were then incubated for 30 h, respectively. The results demonstrated that in WT BMDMs, viral nucleoprotein (NP) expression was comparable between the control and Korean red ginseng-treated groups for both HSV-1 and VACV. Similarly, in *Zbp1*^–/–^ BMDMs, the levels of viral NP expression were consistent with those observed in WT BMDMs (Fig. S3A-F). These findings indicate that ZBP1-mediated cell death does not play a significant role in the response to HSV-1 or VACV infection. This suggests that Korean Red ginseng did not demonstrate efficacy of viral expression against these DNA viruses under the experimental conditions.

### ZBP1 is as a crucial innate immune sensor of Influenza A virus

ZBP1, also known as the DNA-dependent activator of interferon regulatory factor (DAI), is a critical non-NLR family inflammasome sensor integral to viral detection. This cytoplasmic sensor is essential for the recognition and response to viral pathogens, particularly IAV (Karki et al., 2022; Kuriakose et al., 2016; Thapa et al., 2016). Upon binding to viral RNA, ZBP1 initiates a signaling cascade resulting in the activation of inflammatory cytokines, including type I interferons (Karki et al., 2022). This mechanism involves the formation of inflammasomes within primary murine BMDMs. Inflammasome activation subsequently leads to the production of pro-inflammatory cytokines, which are crucial for orchestrating an effective antiviral response. Furthermore, the ability of ZBP1 is to mediate programmed cell death pathways, such as PANoptosis, highlights its significant role in controlling viral infections and restricting viral dissemination (Banoth et al., 2020; Karki et al., 2022; Kesavardhana et al., 2020; Lee et al., 2021b; Oh & Lee, 2023).

**Fig. 2.**
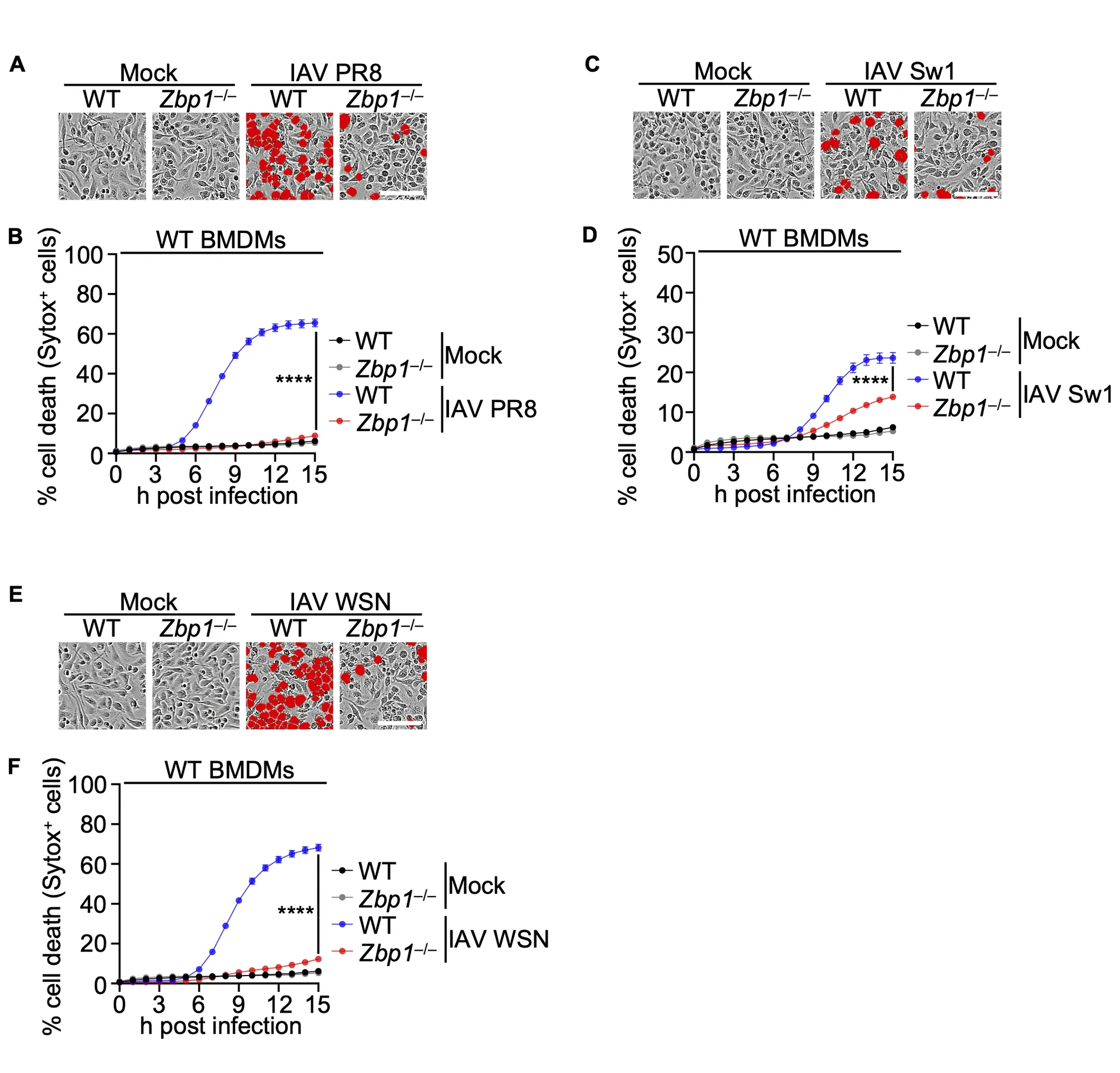
Cell death induced by IAV infection depends on ZBP1. (A) Cell death in wild-type (WT) or *Zbp1*^–/–^ bone marrow-derived macrophages (BMDMs) after IAV PR8 infection for 15 h. Red indicates dead cells. Scale bar, 100 μm. Images are representative of at least three independent experiments. (B) Quantification of the cell death in panel A. Data are mean±s.e.m. ^****^P<0.0001 (two-way ANOVA with Dunnett’s multiple comparisons test; n=9 from three biologically independent samples). (C) Cell death in WT or *Zbp1*^–/–^ BMDMs after IAV Switzerland/2013 infection for 15 h. Red indicates dead cells. Scale bar, 100 μm. Images are representative of at least three independent experiments. (D) Quantification of the cell death in panel C. Data are mean±s.e.m. ^****^P<0.0001 (two-way ANOVA with Dunnett’s multiple comparisons test; n=9 from three biologically independent samples). (E) Cell death in WT or *Zbp1*^–/–^ BMDMs after IAV WSN infection for 15 h. Red indicates dead cells. Scale bar, 100 μm. Images are representative of at least three independent experiments. (F) Quantification of the cell death in panel E. Data are mean±s.e.m. ^****^P<0.0001 (two-way ANOVA with Dunnett’s multiple comparisons test; n=9 from three biologically independent samples).

Subsequently, in our investigation, we examined the impact of ZBP1 deficiency on IAV infection by infecting *Zbp1*^–/–^ BMDMs alongside WT BMDMs with IAV strains. Before that, among the various strains of Influenza A virus, PR8, Switzerland/2013, and WSN were selected as criteria for causing high host cell death in WT BMDMs. Results confirmed a significant reduction in cell death in *Zbp1*^–/–^ BMDMs compared to their WT BMDMs (Fig. 2A-F).

### ZBP1 and Korean Red ginseng induce cell death thereby it reduces various influenza viral protein expression

**Fig. 3.**
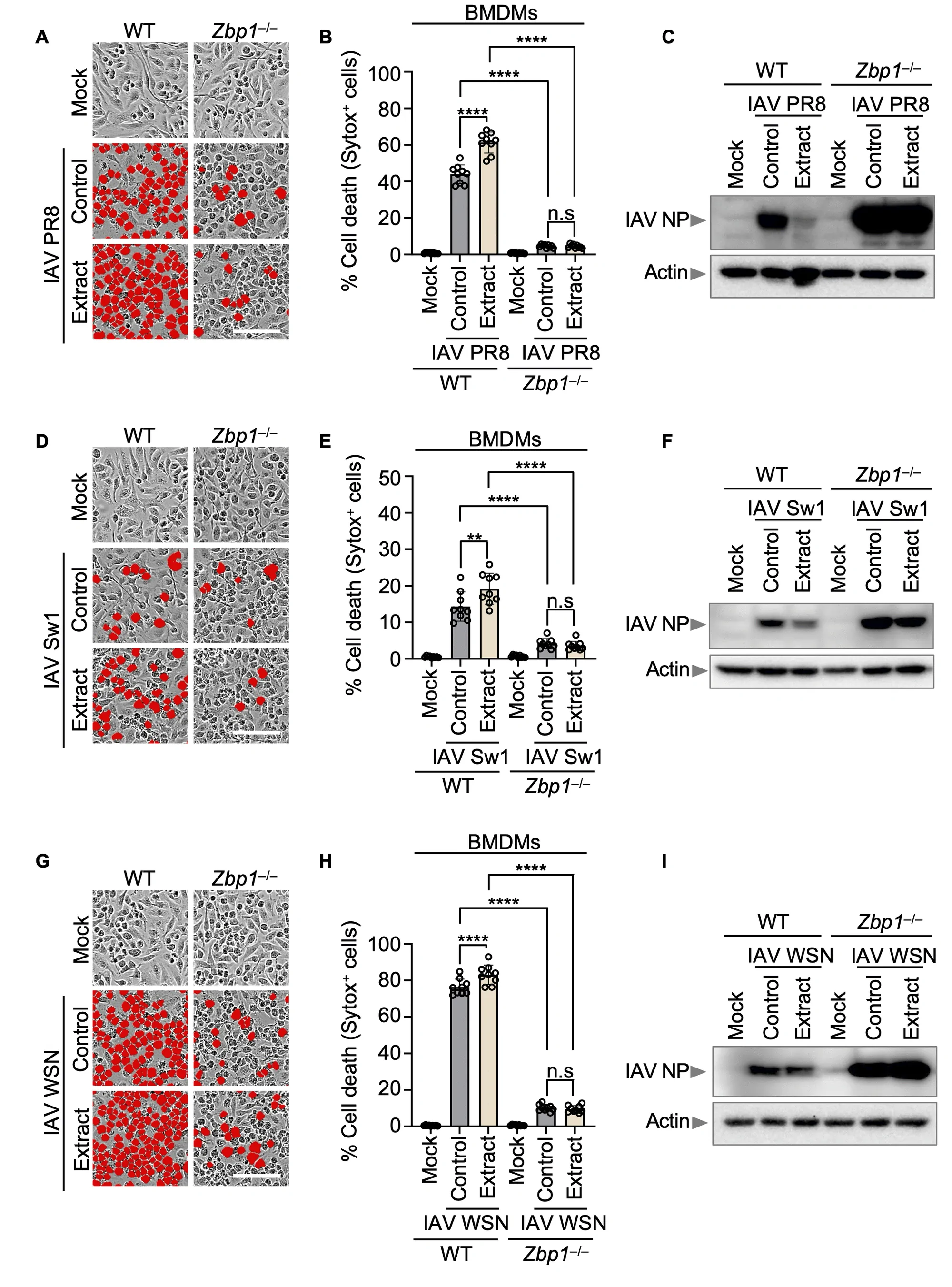
ZBP1 reduces the amount of IAV viral protein expression during IAV infection with Korean Red ginseng extracts. (A) Cell death in wild-type (WT) and *Zbp1*^–/–^ bone marrow-derived macrophages (BMDMs) after IAV PR8 infection and Korean Red ginseng extracts treatment for 30 h. Red indicates dead cells. Scale bar, 100 μm. (B) Quantification of the cell death in panel A. Data are mean±s.e.m. ^****^P<0.0001; ns, not significant (one-way ANOVA with Dunnett’s multiple comparisons test; n=9 from four biologically independent samples). (C) Immunoblot analysis of IAV NP and actin expression in WT and *Zbp1*^–/–^ BMDMs after IAV PR8 infection and Korean Red ginseng extracts treatment for 30 h. Data are representative of at least three independent experiments. (D) Cell death in WT and *Zbp1*^–/–^ BMDMs after IAV Switzerland/2013 infection and Korean Red ginseng extracts treatment for 30h. Red indicates dead cells. Scale bar, 100 μm. (E) Quantification of the cell death in panel C. Data are mean±s.e.m. ^****^P<0.0001; *^*^P<0.01; ns, not significant (one-way ANOVA with Dunnett’s multiple comparisons test; n=9 from four biologically independent samples). (F) Immunoblot analysis of IAV NP and actin expression in WT and *Zbp1*^–/–^ BMDMs after IAV Switzerland/2013 infection and Korean Red ginseng extracts treatment for 30 h. Data are representative of at least three independent experiments. (G) Cell death in WT and *Zbp1*^–/–^ BMDMs after IAV WSN infection and Korean Red ginseng extracts treatment for 30 h. Red indicates dead cells. Scale bar, 100 μm. (H) Quantification of the cell death in panel G. Data are mean±s.e.m. ^****^P<0.0001; ns, not significant (one-way ANOVA with Dunnett’s multiple comparisons test; n=9 from four biologically independent samples). (I) Immunoblot analysis of IAV NP and actin expression in WT and *Zbp1*^–/–^ BMDMs after IAV WSN infection and Korean Red ginseng extracts treatment for 30 h. Data are representative of at least three independent experiments.

To investigate the relationship between Korean Red ginseng and ZBP1 in influenza virus infection. we infected IAV 3 strains (PR8, WSN, and Switzerland/2013) in both WT BMDMs and *Zbp1*^–/–^ BMDMs at a MOI of 10. One-hour post-infection, FBS was added to inhibit viral entry, followed by the administration of Korean Red ginseng extract at a concentration of 250 μg/ml. Both cells were then incubated for an additional 30 h. In WT BMDMs, Korean Red ginseng treatment group resulted in a marked increase in cell death compared to the control group, while the expression level of the influenza A viral NP was notably reduced. Conversely, in *Zbp1*^–/–^ BMDMs, both the virus-only and virus-Korean Red ginseng treated groups demonstrated significantly lower levels of cell death relative to WT BMDMs. Additionally, NP expression was substantially higher in *Zbp1*^–/–^ BMDMs than in WT BMDMs. We found that Korean Red ginseng is associated with ZBP1 in inducing cell death, thereby reducing the expression of various influenza viral proteins (Fig. 3A-I).

### ZBP1 is required for host defense thereby it reduces the expression of influenza viral protein NP along with Korean Red ginseng

**Fig. 4.**
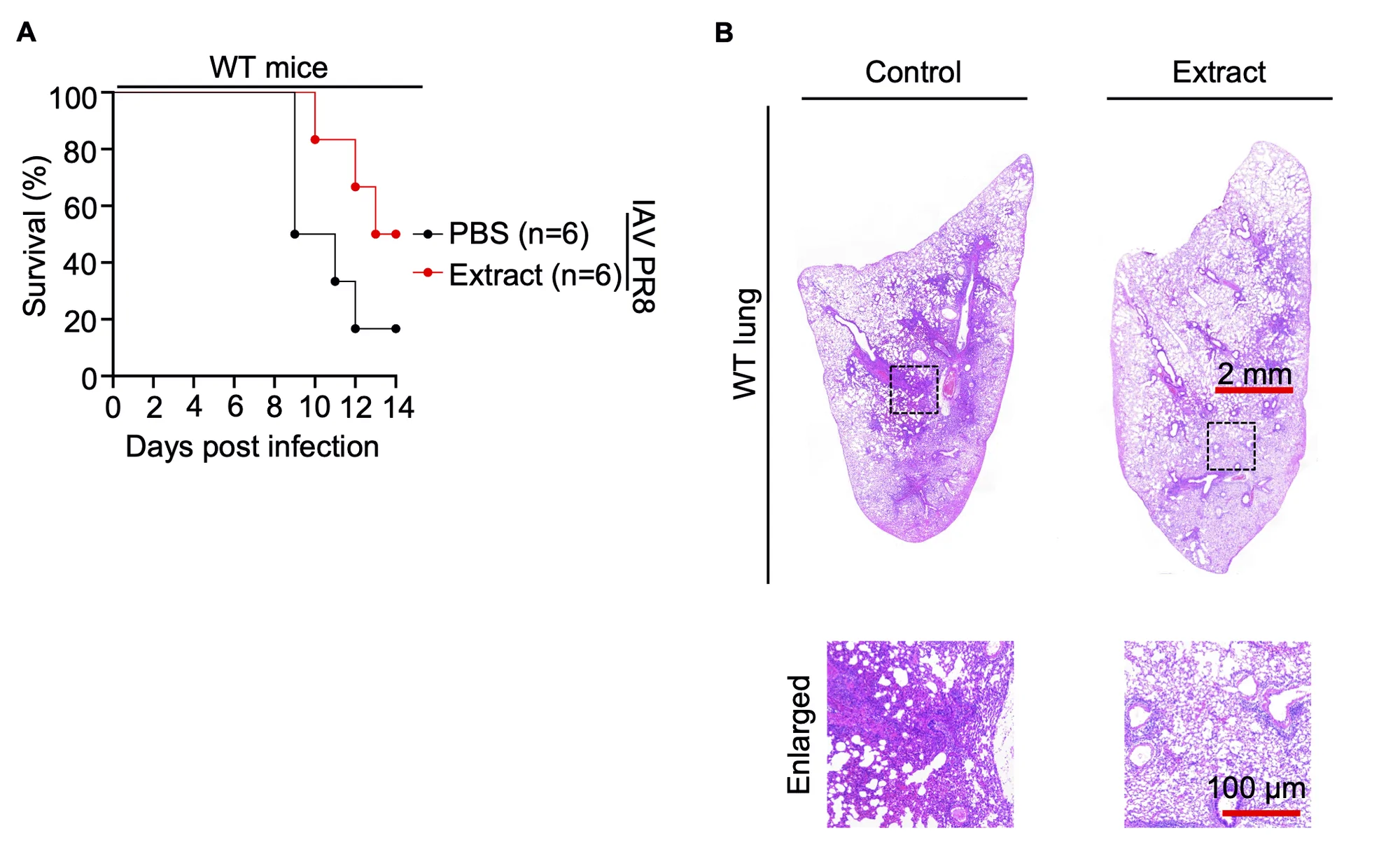
Korean Red ginseng reduces mortality against IAV PR8 in WT mice. (A) Survival of 6- to 8-week-old wild-type (WT) mice treated with PBS (n=6) or Korean Red ginseng extracts (n=6) every day for 7 days before IAV PR8 infection and for 14 days after IAV PR8 infection. *P* values for survival of Korean Red ginseng extracts group versus PBS group are shown in the key. *P*=0.1069 (log-rank (Mantel-Cox) test). (B) Lung tissue sections of WT mice were stained with hematoxylin and eosin. Scale bar, 2 mm (top), 100 μm (bottom).

Subsequently, WT mice and *Zbp1*^–/–^ mice were administered Korean Red ginseng for a duration of 7 days, after which they were inoculated with Influenza A virus PR8 on the 7th day. The mice were then closely monitored for changes in weight and overall survival over a span of 14 days. Experimental data show that in WT mice, treatment with Korean Red ginseng extracts improve survival compared to the control group treated with the virus alone (Fig. 4A). However, in *Zbp1*^–/–^ mice, no significant difference in survival was observed between the virus-treated control group and Korean Red ginseng-treated group. These findings suggest that the beneficial effects of Korean Red ginseng on survival in IAV-infected mice are mediated through the ZBP1 pathway. In the absence of ZBP1, Korean Red ginseng fails to enhance survival, indicating that ZBP1 is essential for the activation of cell death pathways that contribute to the antiviral response facilitated by Korean Red ginseng. This result highlights the critical role of ZBP1 in the immunomodulatory effects of Korean Red ginseng and its potential as a therapeutic agent (Fig. S4A). Histopathological analysis of lung tissue from WT mice revealed that those administered red ginseng exhibited significantly reduced inflammation compared to those solely infected with the virus (Fig. 4B). On the other hand, histopathological analysis of the lungs in *Zbp1*^–/–^ mice revealed that both the control and extract-treated groups exhibited similar patterns to those observed in the control of WT mice. This finding indicates that *Zbp1*^–/–^ mice, when infected with the influenza virus, are unable to induce ZBP1-mediated cell death. Consequently, we concluded that administration of Korean Red ginseng enhancing ZBP1-mediated cell death did not cause additional observable changes in the *Zbp1*^–/–^ mouse model (Fig. S4B).

## Discussion

Red ginseng is derived from the roots of the Panax plant, it undergoes a specialized preparation process involving steaming and drying, enhancing its nutritional properties. Traditionally, Red ginseng used as a drug for viral disease or medicine to enhance the immune system (Kiefer & Pantuso, 2003). The presence of ginsenosides, unique active compounds, is thought to play a pivotal role in these immunomodulatory effects (Liu & Fan, 2018; Qu et al., 2011; Riaz et al., 2019). However, there were no medical evidence to prove the effectiveness. Current studies revealed that during viral infection, intake of ginseng is helpful to activating immune system and reducing viral replication by *in vitro* and *in vivo* experiments (Lee et al., 2014b, 2015). As research continues to unravel the complex interplay between ginseng and the immune system, the potential for ginseng-derived compounds to become recognized therapeutic agents in modern medicine becomes increasingly promising, blending ancient wisdom with contemporary scientific exploration (Liu & Fan, 2018; Riaz et al., 2019; Wang et al., 2021b; Xu et al., 2020; Yoon et al., 2023). However, there was still a lack of understanding regarding the molecular mechanisms involved in activating the immune system.

ZBP1 is a protein encoded by the ZBP1 gene. This protein plays a crucial role in the immune system, particularly in the detection of viral infections and the subsequent activation of antiviral responses. ZBP1 can detect viral nucleic acids, such as Z-RNA and Z-DNA, which are often produced during viral infections like IAV (Zhang et al., 2020). Z-RNA is a left-handed helical form of RNA distinguished by its unique zigzag sugar-phosphate backbone and alternating anti and syn conformations of nucleotides (Krall et al., 2023). This structural peculiarity sets it apart from the more prevalent right-handed RNA structures (Bartas et al., 2022). Notably, Z-RNA serves as a crucial molecular pattern recognized by proteins like ZBP1, initiating immune signalling cascades upon viral infection. As a cytosolic nucleic acid sensor, ZBP1 can recognize and bind to specific DNA structures, including Z-DNA, which can form during viral infections. The presence of Z-DNA or other abnormal DNA structures serves as a sign of viral activity and triggers ZBP1-mediated immune responses (Jiao et al., 2020; Lee et al., 2021c; Li et al., 2023).

Once ZBP1 detects IAV-derived RNA, it initiates a signaling cascade that activates the immune system's response. ZBP1 acts as an early warning signal, triggering downstream pathways that lead to the production of inflammatory cytokines and the activation of immune cells. PANoptosis is a unique form of programmed cell death characterized by the integration of pyroptosis, apoptosis, and necroptosis pathways. Pyroptosis involves inflammatory cell death mediated by inflammasomes, leading to cytokine release and cell lysis (Cookson & Brennan, 2001; Martinon et al., 2002). Apoptosis is a controlled form of cell death that occurs in response to various signals, resulting in cellular dismantling without inflammation (Boldin et al., 1996; Gross et al., 1999; Kim et al., 2005; Li et al., 1997, 1998; Luo et al., 1998; Muzio et al., 1996; Zou et al., 1997). Necroptosis is a regulated form of necrosis driven by receptor-interacting protein kinases (RIPK1 and RIPK3), leading to cell swelling and membrane rupture (Dhuriya & Sharma, 2018; Gong et al., 2019; Murphy et al., 2013; Nailwal & Chan, 2019; Newton et al., 2019; Sun et al., 2012; Zhao et al., 2012). PANoptosis combines aspects of these pathways to orchestrate a coordinated cell death response to diverse stimuli, ensuring effective clearance of damaged cells and pathogens (Karki et al., 2021b; Lee et al., 2020, 2021a; Oh & Lee, 2023; Wang et al., 2022b).

The role of ZBP1 in IAV detection and immunity is crucial for several reasons. Firstly, it provides an extra layer of defense against IAV by recognizing abnormal DNA structures that may be generated during viral replication. Secondly, the initiation of PANoptosis helps to rapidly contain and eradicate the infection, limiting the virus's ability to replicate and spread to neighboring cells and tissues. Finally, the activation of inflammatory responses by ZBP1 contributes to the overall immune defense against IAV and potentially other pathogens (Karki et al., 2022; Thomas et al., 2020). ZBP1 is also related to inflammasome. The inflammasome is a critical component of the innate immune system, serving as a sensor and signalling platform that detects danger signals and initiates inflammatory responses through the processing of pro-inflammatory cytokines (Yu et al., 2024). Its activation plays a crucial role in defending against pathogens, promoting tissue repair, and maintaining immune homeostasis.

In previous studies, there have been many studies to prove the antiviral activity of red ginseng against various viruses (Baek et al., 2010; Chandra Das et al., 2023; Cho et al., 2023; Lee et al., 2013; Pei et al., 2011; Yang et al., 2018). In particular, there have been studies that have investigated whether taking red ginseng before and after an influenza virus infection can help people overcome viral infection. Korean Red ginseng extracts increase the immunomodulatory cytokines and pro-inflammatory cytokines during different strains of influenza virus infection in in-vitro experiments (Lee et al., 2014a; Park et al., 2014; Yoo et al., 2012a, 2012b). And on the in vivo experiments, ginseng treated mice group resulted in higher survival than control groups during infection of IAV (Kim et al., 2016, 2011). However, most of the existing studies have only shown that the treatment of cells or mice with red ginseng can inhibit viral replication. There is lack of studies about molecular mechanisms that how to regulate the immune activation.

In this study, Korean Red ginseng extracts treatment during IAV infection increase the cell death rate and lower the viral expression of cell (Fig. 1). When infected with IAV, to protect the host, innate immunity is activated by sensing the viral genome by ZBP1. As a part of innate immunity, programmed cell death occurs to reduce the amount of viral particle and prevent the viral replication. Thus, in the absence of ZBP1, cell death for host defence does not occur. This is not specific to IAV A/Puerto Rico/8/34 but also to various strains of IAV, including A/Switzerland/9715293/2013, and A/WSN/1933 (Fig. 2). And induced cell death reduces the number of viral proteins in cell (Fig. 3). Therefore, Korean Red ginseng is used as immune enhancer of ZBP1 mediated cell death process. Applying this result to mice as well, wild-type (WT) mice treated with Korean Red ginseng extracts group resulted higher survival than control group. But in the *Zbp1*^–/–^ mice group, there are no significant difference in between control group (Fig. 4 and Fig. S4). In the absence of ZBP1, it does not induce programmed cell death. Therefore, the treatment with Korean Red ginseng did not eliminate the virus, as it could not cause cell death due to the absence of ZBP1, as demonstrated in the in vivo experiment.

This study elucidates an indirect connection between ZBP1 and Korean Red ginseng extracts. ZBP1, sensor of IAV viral DNA, modulate the cell death during IAV infection. And Korean Red ginseng extracts effectively reduce the viral protein of IAV. From this, we can infer that ZBP1 and Korean Red ginseng extracts interact with each other to defend the host during IAV infection. Then Korean Red ginseng extracts can be additional immune enhancer in context of IAV infection. Our findings establish a foundation for further investigation into red ginseng-derived compounds or analogs with enhanced immunomodulatory properties. The intricate relationship between red ginseng and the ZBP1 protein presents promising opportunities for future research and therapeutic innovation. A primary focus for subsequent studies will be to thoroughly elucidate the mechanisms by which Korean Red ginseng interacts with ZBP1 and influences the programmed cell death pathway, a complex process involving multiple cellular mechanisms.

Advanced molecular techniques will be crucial in uncovering the precise pathways through which Korean Red ginseng modulates ZBP1 function, thereby facilitating the development of targeted red ginseng-derived compounds. Moreover, the role of Korean Red ginseng in regulating PANoptosis, particularly in the context of viral infections, warrants further investigation. Future research should explore potential synergistic interactions between Korean Red ginseng and conventional antiviral therapies to enhance therapeutic efficacy and minimize adverse effects.

## Supplementary Information

Fig. S1. 250 μg/mL of Korean Red ginseng is proper concentration that does not induce cell death in BMDMs. A Cell death in wild-type (WT) bone marrow-derived macrophages (BMDMs) after Korean Red ginseng extracts treatment for 30h with does dependent manner. Red indicates dead cells. Scale bar, 100μm. B Quantification of the cell death in panel A. Data are mean±s.e.m. ^***^*P*<0.001 (one-way ANOVA with Dunnett’s multiple comparisons test; n=4 from three biologically independent samples). C Cell death in A549 treated with Korean Red ginseng extracts treatment for 30h with does dependent manner. Red indicates dead cells. Scale bar, 100μm. D Quantification in the cell death in panel C. Data are mean±s.e.m. ns, not significant (one-way ANOVA with Dunnett’s multiple comparisons test; n=9 from three biologically independent samples).

Fig. S2. Korean Red ginseng extracts induce no changes of cell death and viral protein expression during other RNA viral infections; Human coronavirus-NL63 and Mumps virus. A Cell death in A549 after HCoV-NL63 infection and Korean Red ginseng extracts treatment for 30h. Red indicates dead cells. Scale bar, 100μm. Images are representative of at least three independent experiments. B Quantification of the cell death in panel A. Data are mean±s.e.m. ns, not significant (one-way ANOVA with Dunnett’s multiple comparisons test; n=9 from three biologically independent samples). C Immunoblot analysis of HCoV-NL63 NP expression in A549 after HCoV-NL63 infection and Korean Red ginseng extracts treatment. Data are representative of at least three independent experiments. D Cell death in A549 after mumps virus (MuV) infection and Korean Red ginseng extracts treatment for 30h. Red indicates dead cells. Scale bar, 100μm. Images are representative of at least three independent experiments. E Quantification of the cell death in panel D. Data are mean±s.e.m. ns, not significant (one-way ANOVA with Dunnett’s multiple comparisons test; n=9 from three biologically independent samples). F Immunoblot analysis of MuV NP expression in A549 after MuV infection and Korean Red ginseng extracts treatment. Data are representative of at least three independent experiments.

Fig. S3. Korean Red ginseng does not reduce the expression of viral protein during DNA virus infection; Herpes Simplex virus-1 or Vaccinia virus. A Cell death in wild-type (WT) and *Zbp1*^–/–^ bone marrow-derived macrophages (BMDMs) after herpes simplex virus-1 (HSV-1) infection and Korean Red ginseng extracts treatment for 30h. Red indicates dead cells. Scale bar, 100μm. Images are representative of at least three independent experiments. B Quantification of the cell death in panel A. Data are mean±s.e.m. ns, not significant (one-way ANOVA with Dunnett’s multiple comparisons test; n=9 from three biologically independent samples). C Immunoblot analysis of HSV-1 ICP8 and actin expression in WT and *Zbp1*^–/–^ BMDMs after HSV-1 infection and Korean Red ginseng extracts treatment for 30h. Data are representative of at least three independent experiments. D Cell death in wild-type (WT) and *Zbp1*^–/–^ bone marrow-derived macrophages (BMDMs) after vaccinia virus (VACV) infection and Korean Red ginseng extracts treatment for 30h. Red indicates dead cells. Scale bar, 100μm. Images are representative of at least three independent experiments. E Quantification of the cell death in panel D. Data are mean±s.e.m. ^*^*P*<0.05 (one-way ANOVA with Dunnett’s multiple comparisons test; n=9 from three biologically independent samples). F Immunoblot analysis of VACV and actin expression in WT and *Zbp1*^–/–^ BMDMs after VACV infection and Korean Red ginseng extracts treatment for 30h. Data are representative of at least three independent experiments.

Fig. S4. Korean Red ginseng cannot counteract influenza virus due to the absence of ZBP1. A The survival of 6- to 8-week-old *Zbp1*^–/–^ mice treated with PBS (n=6) or Korean Red ginseng extracts (n=7) every day for 7 days before IAV PR8 infection and for 14 days after IAV PR8 infection. *P* values for survival of Korean Red ginseng extracts group versus PBS group are shown in the key. *P*=0.6526 (log-rank (Mantel-Cox) test). B Lung tissue sections of *Zbp1*^–/–^ mice were stained with hematoxylin and eosin. Scale bar, 2mm (top), 100μm (bottom).
