# Supplementary material for: Korean Red ginseng enhances ZBP1-mediated cell death to suppress viral protein expression in host defense against Influenza A virus
Source: J Microbiol. 2025 Jan 24;63(1):e.2409007. doi: 10.71150/jm.2409007 (PMC13577078; doi:10.71150/jm.2409007)
Supplement: Supplementary file 1 [file jm-2409007-Supplementary-Fig-S1.pdf]

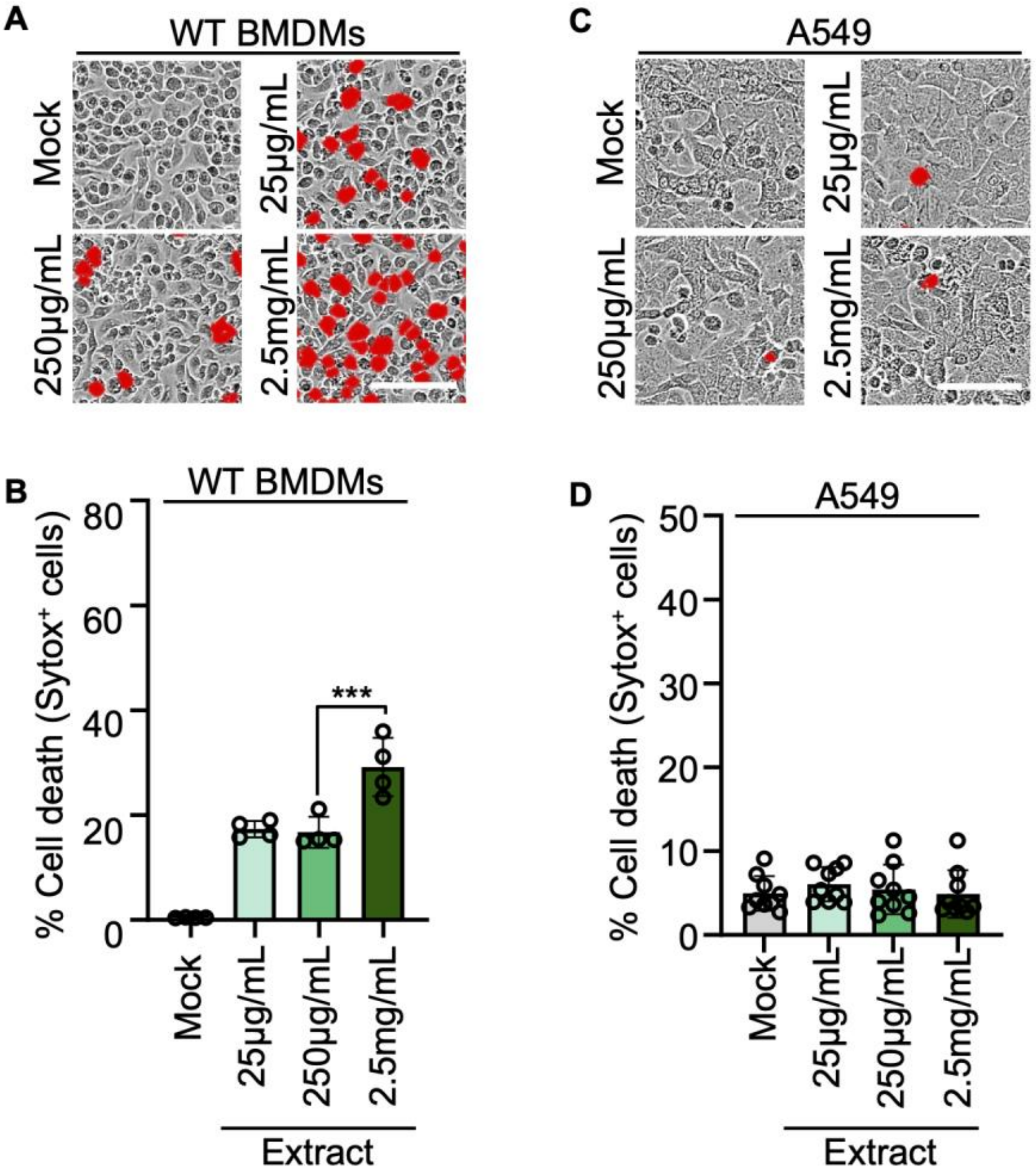

Fig. S1. 250 μg/mL of Korean Red ginseng is proper concentration that does not induce cell death in BMDMs.

A Cell death in wild-type (WT) bone marrow-derived macrophages (BMDMs) after Korean Red ginseng extracts treatment for 30h with does dependent manner. Red indicates dead cells. Scale bar, 100μm. **B** Quantification of the cell death in panel A. Data are mean±s.e.m. ***P<0.001 (one-way ANOVA with Dunnett's multiple comparisons test; n=4 from three biologically independent samples). **C** Cell death in A549 treated with Korean Red ginseng extracts treatment for 30h with does dependent manner. Red indicates dead cells. Scale bar, 100μm. **D** Quantification in the cell death in panel C. Data are mean±s.e.m. ns, not significant (one-way ANOVA with Dunnett's multiple comparisons test; n=9 from three biologically independent samples).
