# Supplementary material for: Korean Red ginseng enhances ZBP1-mediated cell death to suppress viral protein expression in host defense against Influenza A virus
Source: J Microbiol. 2025 Jan 24;63(1):e.2409007. doi: 10.71150/jm.2409007 (PMC13577078; doi:10.71150/jm.2409007)
Supplement: Supplementary file 2 [file jm-2409007-Supplementary-Fig-S2.pdf]

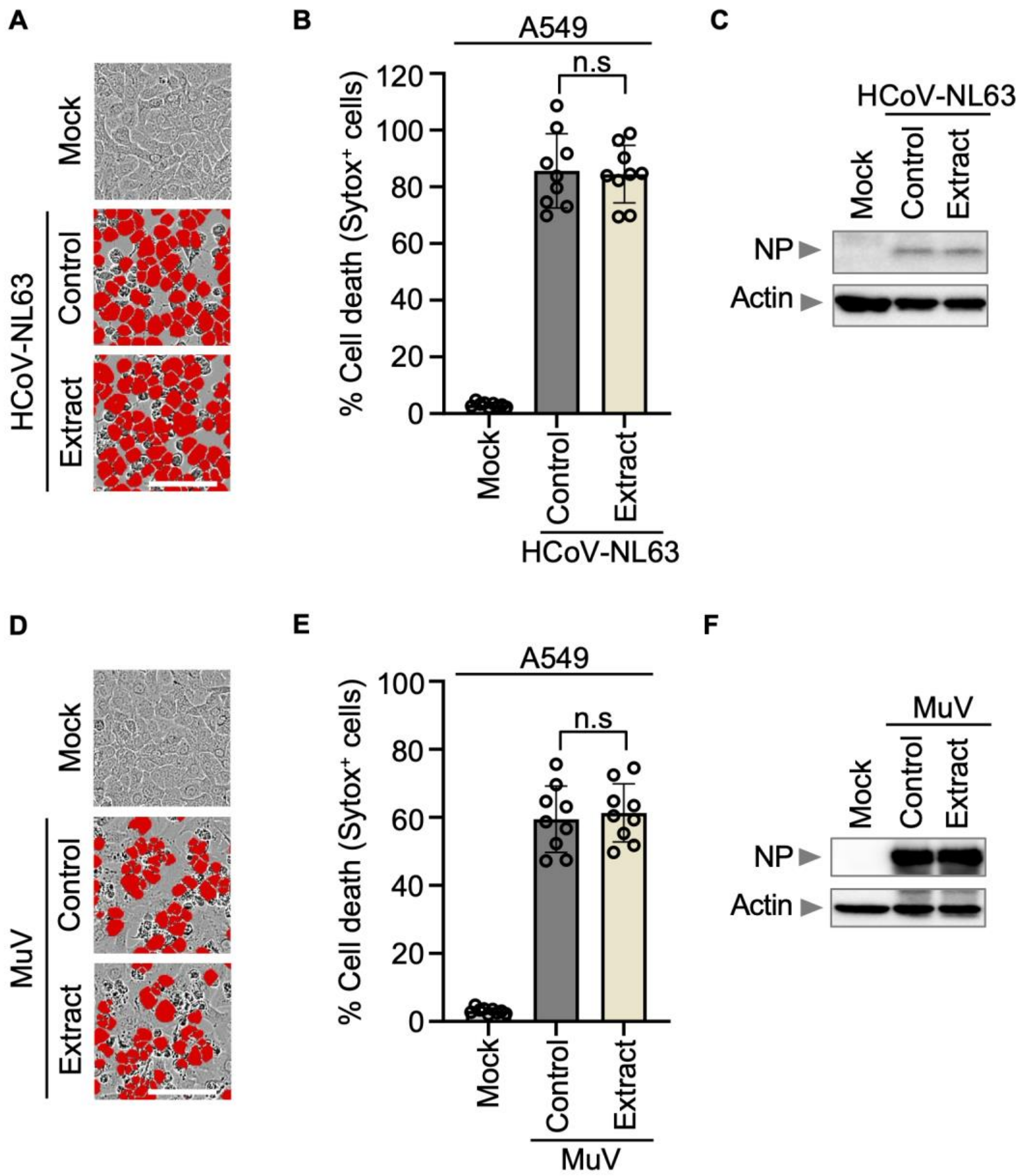

Fig. S2. Korean Red ginseng extracts induce no changes of cell death and viral protein expression during other RNA viral infections; Human coronavirus-NL63 and Mumps virus.

A Cell death in A549 after HCoV-NL63 infection and Korean Red ginseng extracts treatment for 30h. Red indicates dead cells. Scale bar, 100μm. Images are representative of at least three independent experiments. **B** Quantification of the cell death in panel A. Data are mean±s.e.m. ns, not significant (one-way ANOVA with Dunnett's multiple comparisons test; n=9 from three biologically independent samples). **C** Immunoblot analysis of HCoV-NL63 NP expression in

A549 after HCoV-NL63 infection and Korean Red ginseng extracts treatment. Data are representative of at least three independent experiments. **D** Cell death in A549 after mumps virus (MuV) infection and Korean Red ginseng extracts treatment for 30h. Red indicates dead cells. Scale bar, 100μm. Images are representative of at least three independent experiments. **E** Quantification of the cell death in panel D. Data are mean±s.e.m. ns, not significant (one-way ANOVA with Dunnett's multiple comparisons test; n=9 from three biologically independent samples). **F** Immunoblot analysis of MuV NP expression in A549 after MuV infection and Korean Red ginseng extracts treatment. Data are representative of at least three independent experiments.
