# Supplementary material for: Korean Red ginseng enhances ZBP1-mediated cell death to suppress viral protein expression in host defense against Influenza A virus
Source: J Microbiol. 2025 Jan 24;63(1):e.2409007. doi: 10.71150/jm.2409007 (PMC13577078; doi:10.71150/jm.2409007)
Supplement: Supplementary file 3 [file jm-2409007-Supplementary-Fig-S3.pdf]

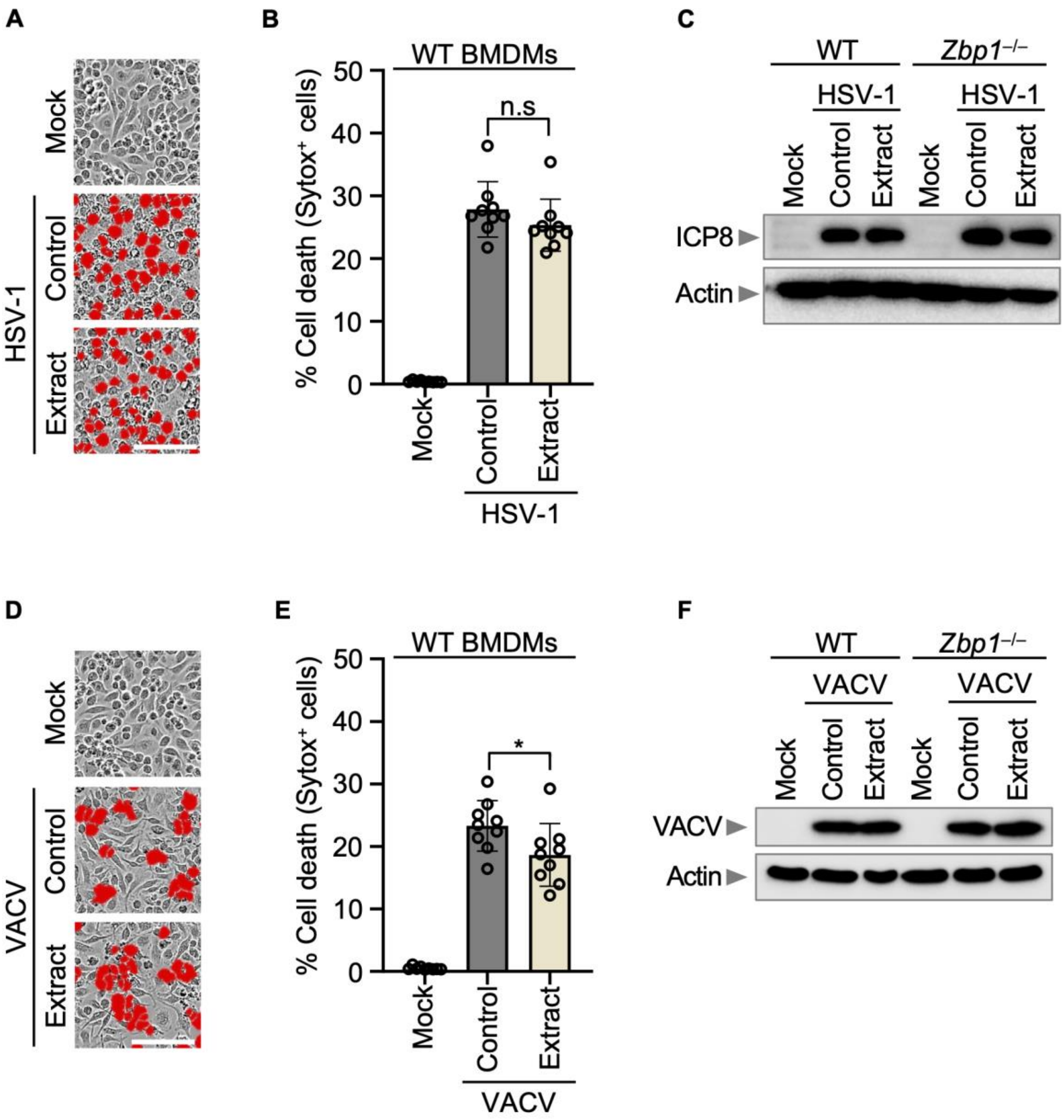

Fig. S3. Korean Red ginseng does not reduce the expression of viral protein during DNA virus infection; Herpes Simplex virus-1 or Vaccinia virus.

A Cell death in wild-type (WT) and *Zbp1*$^{–/–}$ bone marrow-derived macrophages (BMDMs) after herpes simplex virus-1 (HSV-1) infection and Korean Red ginseng extracts treatment for 30h. Red indicates dead cells. Scale bar, 100μm. Images are representative of at least three independent experiments. **B** Quantification of the cell death in panel A. Data are mean±s.e.m. ns, not significant (one-way ANOVA with Dunnett's multiple comparisons test; n=9 from three biologically independent samples). **C** Immunoblot analysis of HSV-1 ICP8 and actin

expression in WT and *Zbp1*$^{-/-}$ BMDMs after HSV-1 infection and Korean Red ginseng extracts treatment for 30h. Data are representative of at least three independent experiments. **D** Cell death in wild-type (WT) and *Zbp1*$^{-/-}$ bone marrow-derived macrophages (BMDMs) after vaccinia virus (VACV) infection and Korean Red ginseng extracts treatment for 30h. Red indicates dead cells. Scale bar, 100μm. Images are representative of at least three independent experiments. **E** Quantification of the cell death in panel D. Data are mean±s.e.m. *$P<0.05$ (one-way ANOVA with Dunnett's multiple comparisons test; n=9 from three biologically independent samples). **F** Immunoblot analysis of VACV and actin expression in WT and *Zbp1*$^{-/-}$ BMDMs after VACV infection and Korean Red ginseng extracts treatment for 30h. Data are representative of at least three independent experiments.
