# Supplementary material for: Korean Red ginseng enhances ZBP1-mediated cell death to suppress viral protein expression in host defense against Influenza A virus
Source: J Microbiol. 2025 Jan 24;63(1):e.2409007. doi: 10.71150/jm.2409007 (PMC13577078; doi:10.71150/jm.2409007)
Supplement: Supplementary file 4 [file jm-2409007-Supplementary-Fig-S4.pdf]

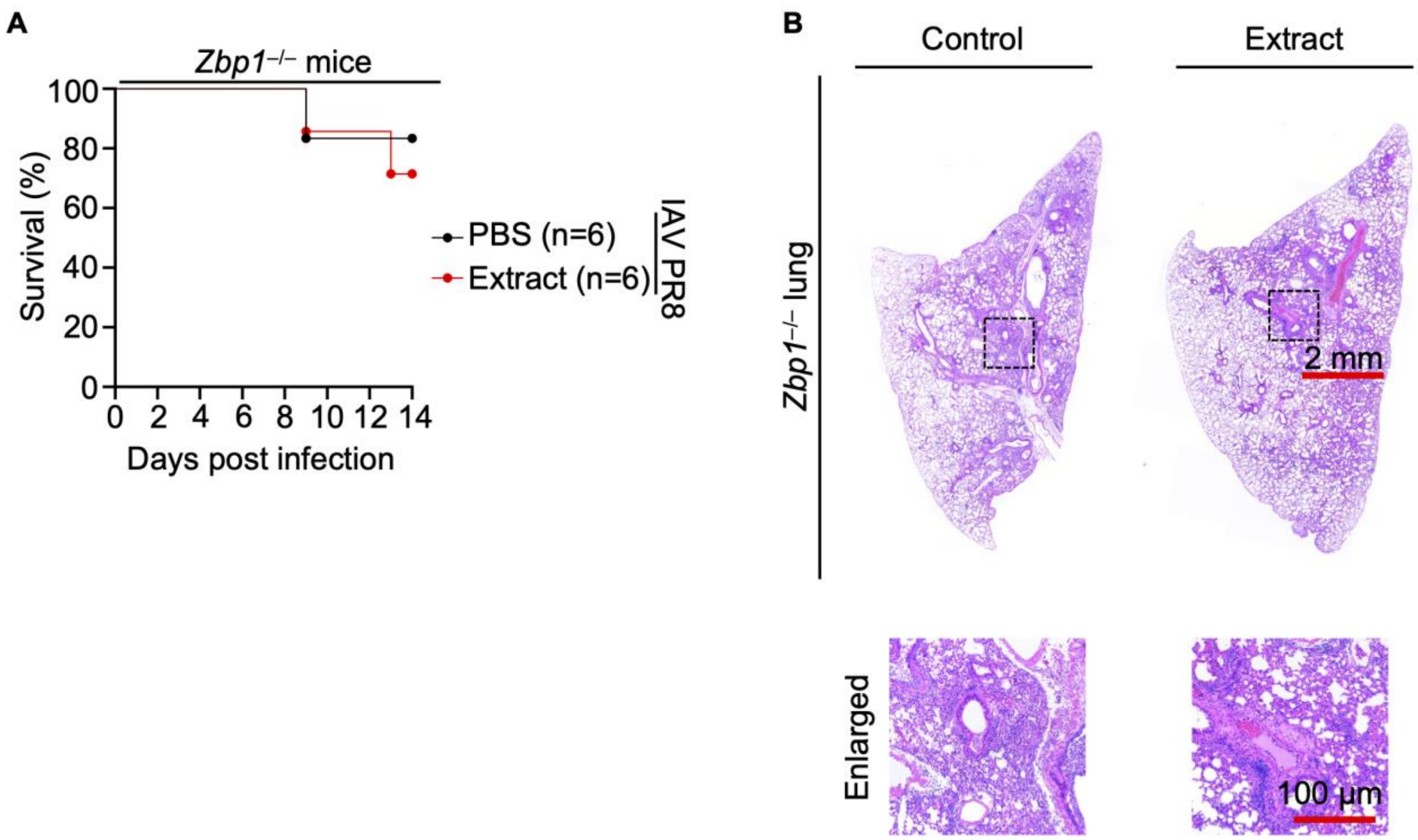

Fig. S4. Korean Red ginseng cannot counteract influenza virus due to the absence of ZBP1.

A The survival of 6- to 8-week-old *Zbp1*$^{-/-}$ mice treated with PBS (n=6) or Korean Red ginseng extracts (n=7) every day for 7 days before IAV PR8 infection and for 14 days after IAV PR8 infection. *P* values for survival of Korean Red ginseng extracts group versus PBS group are shown in the key. *P*=0.6526 (log-rank (Mantel-Cox) test). **B** Lung tissue sections of *Zbp1*$^{-/-}$ mice were stained with hematoxylin and eosin. Scale bar, 2mm (top), 100μm (bottom).
